# Preparation and characterization of a novel nanocomposite based on MnCr-layered double oxide and CoFe_2_O_4_ spinel ferrite for methyl orange adsorption

**DOI:** 10.1038/s41598-023-45136-w

**Published:** 2023-10-21

**Authors:** M. Rekaby, A. I. Abou-Aly, M. El-khatib

**Affiliations:** 1 Department of Physics, Faculty of Science, Alexandria University, Alexandria, Egypt; 2 Department of basic sciences, Faculty of Computer Science and Artificial Intelligence, Pharos University, Alexandria, Egypt

**Keywords:** Materials science, Nanoscience and technology

## Abstract

Herein, the adsorption of methyl orange (MO), a dangerous anionic dye, from an aqueous solution was investigated using a novel magnetic nanocomposite adsorbent. A nanocomposite entitled manganese chromium-layered double oxide/cobalt spinel ferrite, (MnCr)-LDO_5wt.%_/CoFe_2_O_4_, which links the interlayer structural characteristics of layered double oxides (LDOs) with the magnetic properties of spinel ferrites (SFs) was synthesized using the eco-friendly co-precipitation technique. Determination of structural parameters, crystallite size, and micro-strain was done using X-ray diffraction (XRD) analysis. Transmission electron microscopy (TEM) was used to determine grain shape and size. Surface analysis was performed using X-ray photoelectron spectroscopy (XPS) to identify elements and oxidation states present in the prepared nanocomposite. Vibrating sample magnetometer (VSM) was utilized to examine the magnetic characteristic. A comprehensive comparative study about the effectiveness and durability of CoFe_2_O_4_ and (MnCr)_5wt.%_/CoFe_2_O_4_ as nanoadsorbents for MO was conducted. Numerous variables, including contact time, MO concentration, adsorbent dosage, and pH were tested for their effects on the adsorption removal percentages. The findings showed that the maximum removal percentage was 86.1% for 25 ppm of MO was for 0.1 g/100 mL of (MnCr)-LDO_5wt.%_/CoFe_2_O_4_ at pH = 3. Investigations of isotherms and kinetics were conducted under batch conditions. The Langmuir isotherm matched the experimental data, for both nanoadsorbents, quite well due to the homogeneous distribution of active sites. Adsorption kinetics data were found to be compatible with intra-particle diffusion and pseudo-second order models for CoFe_2_O_4_ and (MnCr)_5wt.%_/CoFe_2_O_4_, respectively. A total of five adsorption–desorption cycles were performed to determine the prepared adsorbents’ recyclable nature.

## Introduction

One of the most basic necessities of all living organisms on earth is access to water. As the world’s population grows and the quality of water supplies declines, water is becoming more and more scarce^1^. In addition, various industrial and municipal pollutants are thrown directly into rivers, oceans, and other water sources, harming water resources. Industrial effluents comprise a wide range of poisonous, mutagenic, cancer-causing, and non-recyclable compounds that harm ecosystem health. In addition to polluting water, coloured effluents from dyeing factories also hinder photosynthesis^2^. It is crucial that these contaminants are removed from the water in order to alleviate the rising water issue in the world. One popular and well-known azo anionic dye is methyl orange (MO). The molecules of this water-soluble organic synthetic dye include aromatic and –N=N– groups, which are very poisonous, carcinogenic, and teratogenic^3,4^, as well as being hazardous to the environment and creatures^5,6^. It also has an intense orange colour when dissolved in water. MO is employed as a simulated contaminant in the present study. Some of the processes used to remove water impurities include coagulation, flocculation, membrane filtration, reverse osmosis, ozonolysis, electrostatic precipitation, and adsorption^1–7^. Because of its ease of use and low cost, adsorption is the most widely used technology among the ones mentioned.

Lately, magnetic nanoadsorbent composites are developing as highly effective functional materials with superior micropollutant adsorption and rapid adsorption kinetics^8,9^. In addition to their low-cost synthesis, high surface area, high porosity, high degree of selectivity, binding specificity, and superb reuse ability^8–11^, magnetic nanoadsorbent composites have the ability to be separated immediately from adsorption-remediated waters in the form of magnetic nanoadsorbent-adsorbate sediment by the application of a powerful magnetic field. Layered double oxide/spinel ferrite (LDO/SF) composites are promising candidates as effective magnetic nanoadsorbents. The multilayer structure of LDO and the magnetic nature of SF have been identified as potential properties for a wide range of environmental and industrial applications. As a result, the fabrication of LDO/SF composites is critical, particularly for the adsorption of many pollutants from water, which will be blocked among the layered structure of LDO, and the presence of spinel ferrite will facilitate their easy separation from aqueous solution using only a piece of magnet^8^.

Layered double hydroxides (LDHs) are promising candidates for anionic dye removal due to their surface complexion and electrostatic interactions^11,12^. They are known as hydrotalcite-like compounds that are represented by the formula [M^2+^_(1−z)_ M^3+^_z_ (OH)_2_]^z+^(A^n−^)_z/n_·mH_2_O, where M^2+^, M^3+^ are divalent and trivalent metal cations that result in the indefinite repetition of positively charged sheets (lamellas) alternating with A^n−^ ions (i.e. CO_3_^2−^, OH^−^, Cl^−^, etc.), which are required to balance the net positive charges of the hydroxide layers. The molar ratio z = M^3+^/(M^3+^ + M^2+^) frequently falls between 0.2 and 0.4^10^. Moreover, LDH can be transformed into LDO by calcination at 600 °C, and LDO can be transformed back into LDH after being dissolved in water. This mechanism, known as the memory effect, can increase surface area while decreasing adsorption effectiveness^13^. Layered double oxides (LDOs) have been widely used as anion exchangers, adsorbents, and catalysts^14,15^.

Spinel ferrites are compounds with the general formula MFe_2_O_4_, where M is a divalent metal ion such as Ni^2+^, Co^2+^, Mn^2+^, Zn^2+^, or another metal ion. Cobalt ferrite (CoFe_2_O_4_) is discovered to be more adaptable due to its ferromagnetic feature as well as its high electrochemical stability, making it a significant spinel material. CoFe_2_O_4_ has an inverted spinel structure, which means that ferric ions are present at tetrahedral sites while ferric and cobalt ions are present at octahedral sites. CoFe_2_O_4_ was selected for this investigation because of its high magnetic permeability, low magnetic loss, high cut-off frequency, high saturation magnetization, high curve temperature, temperature stability, low coercivity, and biodegradability^16,17^. Several composites comprised of ferities and LDH/LDO have been conducted^17–20^, including CoFe_2_O_4_/CuAl-LDH, NiFe_2_O_4_/ZnCuCr-LDH, Fe_3_O_4_/Zn–Al–Fe–La-LDH, Fe_3_O_4_/ZnCr-LDH and CoFe_2_O_4_/MgAl-LDO^21^. However, as far as we are aware, no other work has utilized (MnCr)-LDO_5wt.%_/CoFe_2_O_4_ nanocomposites as an affordable adsorbent and active photocatalyst for MO dye removal from aqueous solution.

In this study, a novel magnetic nanocomposite entitled (MnCr)-LDO_5wt.%_/CoFe_2_O_4_ was fabricated through the facile co-precipitation route, and its removal efficiency and photocatalytic activity for methyl orange dye were examined and compared to that of pure CoFe_2_O_4_. Numerous variables, including; the contact time, the dye concentration, the nanoadsorbent dosage, and pH, were tested for their effects on the adsorption rate. Investigations of equilibrium and kinetics were conducted under batch circumstances.

## Experimental technique

### Synthesis

#### Materials

High purity reagents of iron chloride hexahydrate (FeCl_3_.6H_2_O, ≥ 98%, Oxford), cobalt chloride hexahydrate (CoCl_2_·6H_2_O, ≥ 97%, Loba Chemie), manganese chloride tetrahydrate (MnCl_2_·4H_2_O, ≥ 99%, Qualikems), chromium chloride hexahydrate hydrate (CrCl_3_·6H_2_O, 99.9%, Oxford), sodium bicarbonate (Na_2_CO_3_, ≥ 99% Loba Chemie), sodium hydroxide (NaOH, ≥ 98%, Loba Chemie) and methyl orange (C_14_H_14_N_3_NaO_3_S) were utilized. Throughout the synthesis, distilled water was employed as the dispersion solvent.

#### Preparation of MnCr-LDH and MnCr-LDO

By using the low super saturation co-precipitation technique, two aqueous solutions were prepared and double titrated to 10 mL of distilled water while being stirred at 500 rpm. The first solution included 50 mL of distilled water along with 4.4527 g of MnCl_2_·4H_2_O and 1.9983 g of CrCl_3_·6H_2_O. The second solution was for 50 mL of distilled water, 3.3747 g of NaOH, and 2.9809 g of Na_2_CO_3_. After almost three hours, the titration was terminated, and the pH had reached 10. The mixture was then heated to 80 °C for 4 h. The temperature is turned off, and the mixture is aged overnight at room temperature. The solution is then centrifuged at 1000 rpm for 30 min, and the resulting precipitate is washed several times with distillated water until the pH is neutral. The precipitate was dried at 50 °C for 48 h, and finally a calcination process was performed at 600 °C for 3 h to convert the LDH to LDO.

#### ***Preparation of CoFe***_***2***_***O***_***4***_

CoFe_2_O_4_ was synthesized using a simple and environmentally friendly co-precipitation method. Separately, 25 mL of distilled water was used to dissolve 13.515 g of FeCl_3_·6H_2_O and 5.9482 g of CoCl_2_·6H_2_O. The two solutions were mixed and stirred until perfect homogeneity was achieved. The chlorides mixture solution was titrated with 3 M NaOH in 75 mL of distilled water at a very slow rate until the pH reached 13. The mixture is then heated to 80 °C and stirred at 500 rpm for 3 h. A piece of magnet was used to collect the dark brawn precipitate, which was then washed numerous times with distillated water until the pH reached 7. The precipitate was dried at 50 °C overnight before being ground and calcined at 700 °C for 3 h. This method yielded a 6 g CoFe_2_O_4_ sample.

#### ***Preparation of (MnCr)-LDO***_***x***_***/CoFe***_***2***_***O***_***4***_

**Figure 1:**
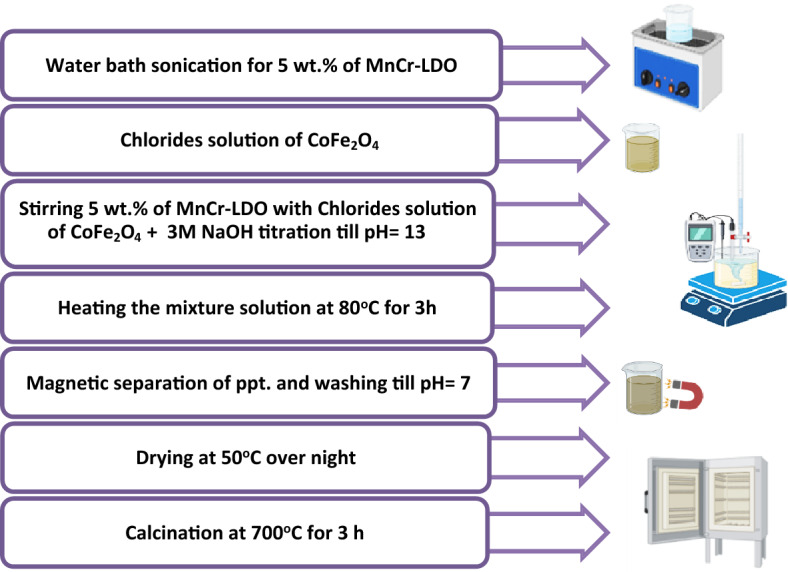
Schematic representation for (MnCr)-LDO_5 wt.%_/CoFe_2_O_4_ preparation steps.

To prepare a composite of type (MnCr)-LDO_x_/CoFe_2_O_4_, 100 mL beaker filled with 25 mL distilled water was used to dissolve the required amount of the prepared MnCr-LDO with a percentage x = 5 wt.% from CoFe_2_O_4_ total weight (6 g), then sonicated for 16 min. The iron and cobalt chloride precursor’s solutions were prepared and stirred at 300 rpm. The MnCr-LDO solution was mixed with the chloride precursor’s solution. Droplets of 3 M NaOH solution were used to make the pH = 13. The mixture was continuously stirred at 500 rpm and heated at 80 °C for 3 h. The precipitate was magnetically separated and washed several times with distilled water to reach neutrality. Drying the precipitate at 50 °C overnight was conducted. After that, the powder was ground and calcined at 700 °C for 3 h. A schematic representation for (MnCr)-LDO_5wt.%_/CoFe_2_O_4_ preparation steps is shown in Fig. 1.

### Characterization

Structural characterization was achieved using the X-ray powder diffraction (XRD) technique with Shimadzu LabX 6100 Japan CuKα radiation (λ = 1.54178 Å). The transmission electron microscope (TEM) model JEOL JEM 1400 Plus was used to observe particle size and sample morphology. The XPS spectra were recorded using the Thermoscientific K-Alpha X-ray photoelectron spectrometer system, with the pass energy constant mode of energy analyzer at 200 eV (full survey XPS spectra) and 50 eV (high-resolution HR-XPS spectra). Al-kα radiation energy source was used under a pressure of 10^−9^ mbar. The vibrational modes were obtained by the FTIR using Bruker Vertex70 Germany (KBr technique-400:4000 wavenumbers). A vibrating sample magnetometer (VSM) model Lake Shore 7410 with a maximum applied magnetic field of 20 kOe was used to conduct the magnetic measurements. Nitrogen adsorption–desorption measurements at 77 K were performed by BELSORB III analyzer, Japan, to determine Brunauer–Emmett–Teller (BET) specific surface area and Barrett-Joyner-Halenda (BJH) average pore diameter. Prior to analysis, samples of about 0.3 g were degassed at 200 °C for 3 h.

### Adsorption test

**Figure 2:**
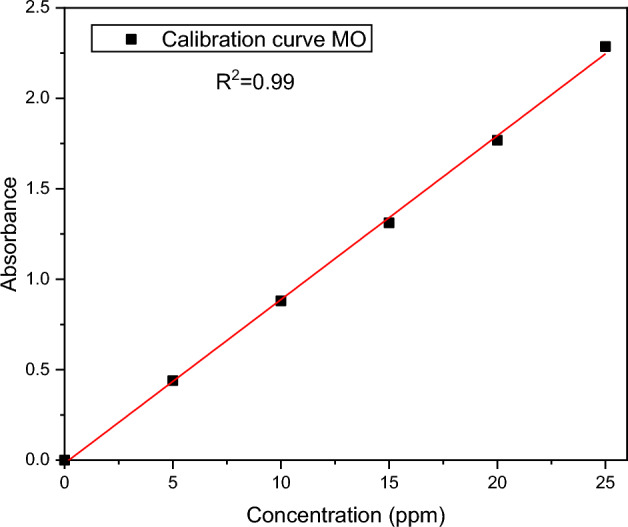
Calibration curve of MO dye.

A standard MO stock solution of 25 ppm was prepared and diluted with distilled water in order to obtain different known initial concentrations, i.e., 5, 10, 15, 20, and 25 ppm. The relative absorbance of these MO concentrations was determined from a UV–Vis spectrophometer (Systronics UV–Vis spectrophometer-117) at a wavelength of 460 nm. Figure 2 depicts the variation of absorbance (A) with MO concentrations. The absorbance is shown to be linearly related to the concentration of MO. Straight line fitting was performed for the experimental data with a correlation factor of R^2^ = 0.99. For equilibrium and kinetic studies, this curve was utilized as a calibration curve to convert absorbance data into concentrations.

The MO adsorption experiments were conducted in batch conditions using two nanoadsorbents termed CoFe_2_O_4_ and (MnCr)-LDO_5wt.%_/CoFe_2_O_4_. Several parameters have been examined to show their effect on MO adsorption. These parameters are the contact time, the dye concentration, the nanoadsorbent dosage, and finally the pH. For the effect of contact time; 25 ppm of MO solutions were kept in 100 mL flasks, and 0.1 g/100 mL of each nanoadsorbent was added to them. The flasks were placed on a shaker with a speed of 150 rpm, and the nanoadsorbents were magnetically separated from the solutions at different contact times (t = 10–150 min). The supernatants were analyzed by UV–Vis spectrophometer, at a wavelength of 460 nm, after each contact time. To study the effect of dye concentration, the dye concentration was varied from 5 to 25 ppm while the nanoadsorbent dosage was fixed at 0.1 g/100 mL, and the contact time was maintained at the equilibrium time of each nanoadsorbent (90 and 120 min for CoFe_2_O_4_ and (MnCr)-LDO_5wt.%_/CoFe_2_O_4_, respectively). The effect of nanoadsorbent dosage was studied by varying the dosage of CoFe_2_O_4_ and (MnCr)-LDO_5wt.%_/CoFe_2_O_4_ from 0.1 g/100 mL to 0.5 g/100 mL, and the rest of the parameters were fixed at 25 ppm MO concentration, and the collection times were their equilibrium times. The effect of pH was conducted with 25 ppm of MO solutions which were agitated with 0.1 g/100 mL of both CoFe_2_O_4_ and (MnCr)-LDO_5wt.%_/CoFe_2_O_4_ at different pH values (3–8) at 25 °C. The dye removal percentage (R %)^22^, the kinetic adsorption capacity of MO dye at equilibrium time, q_e_ (mg L^−1^), and the kinetic adsorption capacity of MO dye at time t, q_t_ (mg L^−1^), were estimated from the following relations^23^:

1 $$R \left( \% \right) = \frac{{C_{o} - C_{e} }}{{C_{o} }} \times 100$$

2 $$q_{e} = \frac{{C_{o} - C_{e} }}{m} \times V$$

3 $$q_{t} = \frac{{C_{o} - C_{t} }}{{C_{o} }} \times 100,$$

where the definition of each parameter in Eqs. (1–3) were tabulated in Table 1. The equilibrium adsorptions of MO dye from aqueous solutions were investigated using Langmuir, Freundlich, and Temkin models for isotherms. Furthermore, the kinetics of MO dye adsorption was examined using Lagergren's pseudo-first order, pseudo-second order, Elovich, and intra-particle diffusion.

**Table 1:** Adsorption parameters.

Symbol	Definition
R (%)	Dye removal percentage
${C}_{o}$	Initial concentrations of MO
${C}_{e}$	Equilibrium concentrations of MO (mg L^−1^)
${C}_{t}$	Is the concentration of MO at a given time t
${q}_{e}$	Kinetic adsorption capacity of MO dye at equilibrium time
${q}_{t}$	Kinetic adsorption capacity of MO dye at time t
V	Dye solution volume (mL)
t	Contact time
m	Adsorbent mass (g)

### Desorption and reusability

At room temperature, 0.1 g/100 mL each of CoFe_2_O_4_ and (MnCr)-LDO_5wt.%_/CoFe_2_O_4_ were added to 100 mL conical flasks with 25 ppm of MO solutions and shaken in an orbital incubator shaker for 90 and 120 min, respectively. The final dye concentration was determined by magnetically separating the mixture. The magnetic nanoadsorbent was recycled by being washed several times with 200 mL distilled water while being stirred constantly for 15 min. After that, a reused magnetic nanoadsorbent was added to a MO solution to initiate adsorption. A total of five adsorption–desorption cycles were performed to determine the material's recyclable nature.

### Photocatalytic test

The degradation of MO solution under UV light irradiation was used to measure the photocatalytic activity of the prepared samples. The reaction mixture was agitated in the dark for 1 h in this experiment to guarantee that MO was adsorbed to saturation on the catalysts. The solutions were then exposed to UV light using a 6 W–365 nm lamp. A 0.1 g photocatalyst was added to a 50 mL MO (5 ppm) solution with a solid/water ratio of 1 g/L. At each irradiation time interval, 5 mL aliquots were extracted and magnetically separated to remove almost all of the catalysts. During the photo-degradation process, the quantities of the leftover dye were detected using UV–Vis spectrophometer by measuring the absorbance of solutions at 460 nm. The identical process was used to carry out the blank reaction, but without inserting the catalyst.

## Results and discussions

### Structural analysis

**Figure 3:**
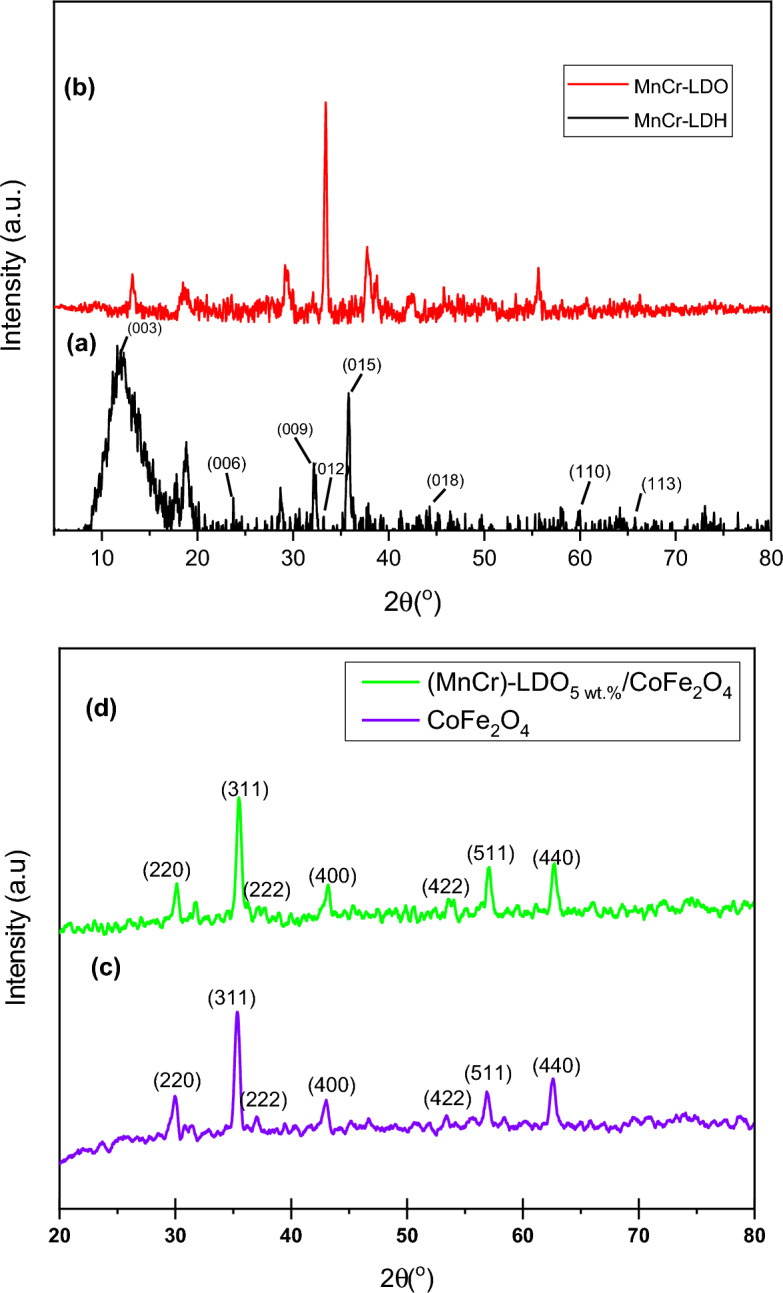
XRD patterns for (**a**) MnCr-LDH, (**b**) MnCr-LDO (**c**) CoFe_2_O_4_ and (**d**) (MnCr)-LDO_5wt.%_/CoFe_2_O_4_.

The XRD patterns for the MnCr-LDH, MnCr-LDO, CoFe_2_O_4_, and (MnCr)-LDO_5wt.%_/CoFe_2_O_4_ samples are shown in Fig. 3a–d, respectively. It is clear that the typical peaks for the hydrotalcite-like crystal structures (JCPDS: 38-0487)^24^ are present in the synthesized MnCr-LDH, as shown in Fig. 3a. These diffraction peaks were well indexed by the hexagonal structure and *R3̅m* rhombohedral symmetry^25^. Specifically, eight peaks occurred at 2θ of 11.97°, 23.65°, 32.82°,33.04°, 38.17°, 45.28°, 55.13 and 65.70°, correspond to (003), (006), (009), (012), (015), (018), (110), and (113) diffraction planes, which are signatures for hydrotalcite-like structures. The basal peaks at (003), (006) and (009) are often connected with the lamellar stacked layers, the (110) correlates with the organizational structure inside the lamellae, and the (012), (015) and (018) are related to the inner layers^26^. Figure 3b is for MnCr-LDO after heating MnCr-LDH at 600 °C for 3 h. The MnCr-LDO is still preserving the hydrotalcite-like crystal structure. The peaks become sharper especially for the basal (003) plane and this reflects the good crystallinity the sample has acquired after calcination. The lattice parameters *a* and *c* of MnCr-LDO were derived from (110) and (009) reflections, respectively, (tabulated in Table 2) according to the hexagonal unit cell symmetry ($a\, = \,b\, \ne \,c; \, \alpha \, = \,\beta \, = \,90^\circ ,\;\gamma \, = \,120^\circ$) using the following equation^27^:

4 $$\frac{1}{{d_{hkl}^{2} }} = \frac{4}{3}\left( {\frac{{h^{2} + hk + k^{2} }}{{a^{2} }}} \right) + \frac{{l^{2} }}{{c^{2} }},$$

where *d*_*hkl*_ is the interplannar spacing. The estimated lattice parameter *a* = 2.8827 Å refers to the distance between the cations inside the structure's layers, whilst the determined value of *c* = 24.5331 Å is connected to layer thickness and interlayer spacing^26^. The lattice parameters values are very close to those estimated by Teixeira et al.^26^ for MgAl-LDO. The average crystallite size *D* of MnCr-LDO has been calculated using Debye–Scherrer equation^28^.

5 $$D = \frac{\alpha \lambda }{{\beta \cos \theta }},$$

where *α* is the Scherrer constant (0.9), *λ* is the X-ray wavelength (1.5406 Å), *β* is full width of half maximum (FWHM) and *θ* is the Bragg’s angle. The micro strain $\varepsilon$ was calculated using the formula suggested in^29^ as follows:

6 $$\varepsilon = \frac{\beta \cos \theta }{4},$$

**Table 2:** Structural parameters (a and c), crystallite size (D), micro strain (ε), TEM particle size (D_TEM_), BET surface area (S_BET_) and average pore diameter (d_P_) for the synthesized samples.

Sample	*a* (Å)	*c* (Å)	*D* (nm)	*ε* × 10^−3^	*D*_*TEM*_ (nm)	*S*_*BET*_ (m^2^/g)	*d*_*P*_ (nm)
MnCr-LDO	2.8827	24.533	27.2	1.09	32	–	–
CoFe_2_O_4_	8.3832	–	18.7	2.15	15.9	27	26
(MnCr)-LDO_5wt.%_/CoFe_2_O_4_	8.4136	–	21.7	2.04	19	30	33

The values of *D* and $\varepsilon$ are listed in Table 2*.* The estimated crystallite size (*D* = 27.2 nm) was larger than that reported for MgAl-LDO by Teixeira et al.^26^ (24.7 nm). Larger crystallite sizes result in narrower peaks, which are associated with more crystallinty, and this appears to be the scenario here. Furthermore, the estimated crystallite size is quite close to that determined for CoCr-LDO by Balayeva et al.^30^ using 0.6 M NaOH. The micro strain $\varepsilon$ value for synthesized MnCr-LDO is found to be 1.09 × 10^−3^.

The XRD pattern of the synthesized CoFe_2_O_4_ (Fig. 3c) was analyzed and compared to standard sheet number JCPDS: 22-1086^31^. Bragg reflections at 2θ of 29.93°, 35.31°, 36.95°, 42.93°, 53.33°, 56.85° and 62.52° correspond to (220), (311), (222), (400), (422), (511), and (440) planes, respectively. This validates the development of ferrite with the FCC structure and the *fd3̅m* space group. Furthermore, the lack of reflections at 2θ = 33°, 49° and 66° revealed that the sample is single phase and devoid of the impurity phase of α-Fe_2_O_3_. Figure 3d displays the XRD pattern of (MnCr)-LDO_5wt.%_/CoFe_2_O_4_ nanocomposite. The resultant nanocomposite inherited high intensity peaks related to CoFe_2_O_4_ and low intensity peaks accounted for MnCr-LDO indicating their successful incorporation. The lattice parameters of CoFe_2_O_4_ and (MnCr)-LDO_5wt.%_/CoFe_2_O_4_ were calculated according to the FCC unit cell based on the following equation^31^

7 $$a = d_{hkl} \sqrt {h^{2} + k^{2} + l^{2} } .$$

The average crystallite sizes and micro strains for CoFe_2_O_4_ and (MnCr)-LDO_5wt.%_/CoFe_2_O_4_ were calculated in the same manner discussed for MnCr-LDO according to Eqs. (5) and (6), and their values were listed in Table 2. It is obvious, from Table 2, that the addition of MnCr-LDO to CoFe_2_O_4_ has created a nanocomposite with enhanced lattice parameters, crystallite size, and reduced micro strain. The increase in lattice parameter and the reduction in micro strain could be attributed to the agglomeration of MnCr-LDO at the grain boundaries, which results in improved crystallite size that reduces the defects and overall line broadening during synthesis^32^. A similar lattice constant value was also observed by Reddy et al.^33^ and Monisha et al.^31^. For CoFe_2_O_4_; Monisha et al.^31^ have found a crystallite size of 16.7 nm, while Manouchehri et al.^34^ have reported an average crystallite size of 13 nm for CoFe_2_O_4_ nanoparticles coated with dimercaptosuccinic acid. Naseri et al.^35^ reported the crystallite size to be 20 nm for MnFe_2_O_4_ nanoparticles. Moreover, Kombaiah et al.^36^ have reported higher values of 56 nm and 43 nm for CoFe_2_O_4_ prepared by conventional and microwave heating method, respectively.

### TEM and BET analysis

**Figure 4:**
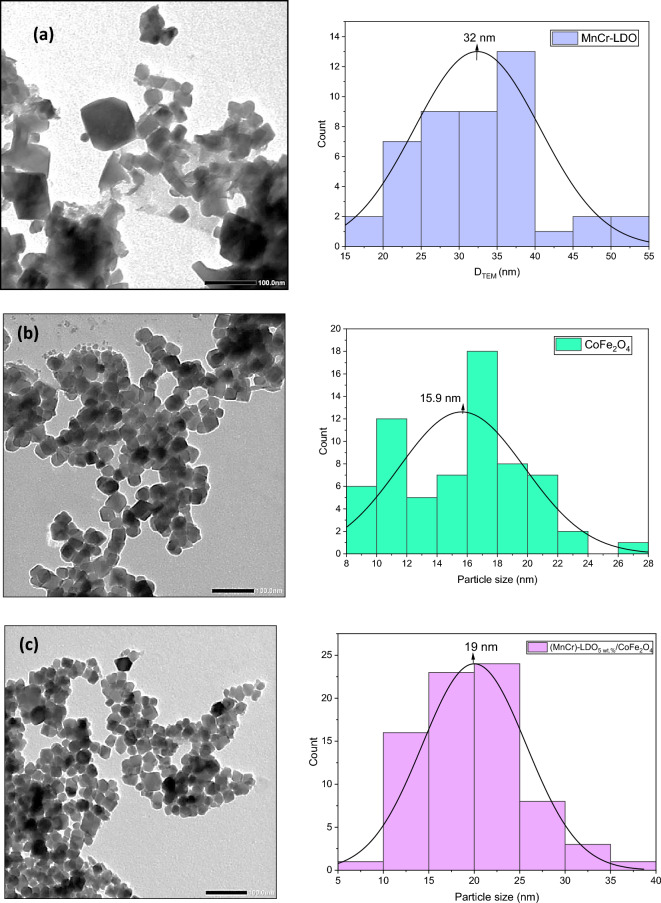
TEM micrographs for (**a**) MnCr-LDO, (**b**) CoFe_2_O_4_, and (**c**) (MnCr)-LDO_5wt.%_/CoFe_2_O_4_.

Figure 4a–c depicts TEM micrographs at scale of 100 nm together with the size distribution histograms of MnCr-LDO, CoFe_2_O_4_, and (MnCr)-LDO_5wt.%_/CoFe_2_O_4_ nanoparticles, respectively. The particle sizes and their distribution were made by “Image J” and Origin Lab software. The average particle sizes, listed in Table 2, were found to be 32 nm, 15.5 nm and 19 nm for MnCr-LDO, CoFe_2_O_4_, and (MnCr)-LDO_5wt.%_/CoFe_2_O_4_ nanoparticles, respectively. These values agree well with the average crystallite sizes estimated from XRD data. It is clear, from Fig. 4a, that MnCr-LDO demonstrated particle morphologies of irregular hexagonal particles. Moreover, a cubic morphologies appeared for CoFe_2_O_4_, and (MnCr)-LDO_5wt.%_/CoFe_2_O_4_ nanoparticles as shown in Fig. 4b,c. Similar cubic particles with size of 25 nm were observed for CoFe_2_O_4_ by Kumar et al.^37^.

**Figure 5:**
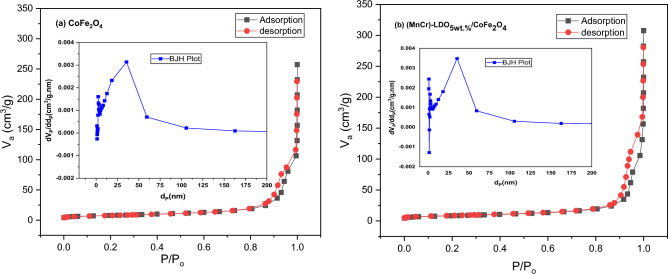
N_2_ adsorption–desorption isotherms and pore size distribution of (**a**) CoFe_2_O_4_, and (**b**) (MnCr)-LDO_5wt.%_/CoFe_2_O_4_.

Figure 5a,b show the obtained BET isotherm and BJH pore size distribution (insets) for CoFe_2_O_4_ and (MnCr)-LDO_5wt.%_/CoFe_2_O_4_ samples, respectively. The surface textural parameters of N_2_ adsorption–desorption analysis were listed in Table 2. The CoFe_2_O_4_ and (MnCr)-LDO_5wt.%_/CoFe_2_O_4_ had BET-specific surface areas (S_BET_) of 27 m^2^/g and 30 m^2^/g, respectively. The (MnCr)-LDO_5wt.%_/CoFe_2_O_4_ composite sample had a BJH adsorption average pore diameter (d_P_) of 33 nm and a pore volume of 0.2264 cm^3^/g, which were greater than the pure CoFe_2_O_4_ sample, which had a d_P_ of 26 nm with a pore volume of 0.1654 cm^3^/g. Similar value of pore diameter was observed by Deng et al.^21^ for CoFe_2_O_4_/MgAl-LDO. As a result, the (MnCr)-LDO_5wt.%/_CoFe_2_O_4_ composite is more effective nanoadsorbent for MO adsorption rather than pure CoFe_2_O_4_.

### FTIR and VSM analysis

**Figure 6:**
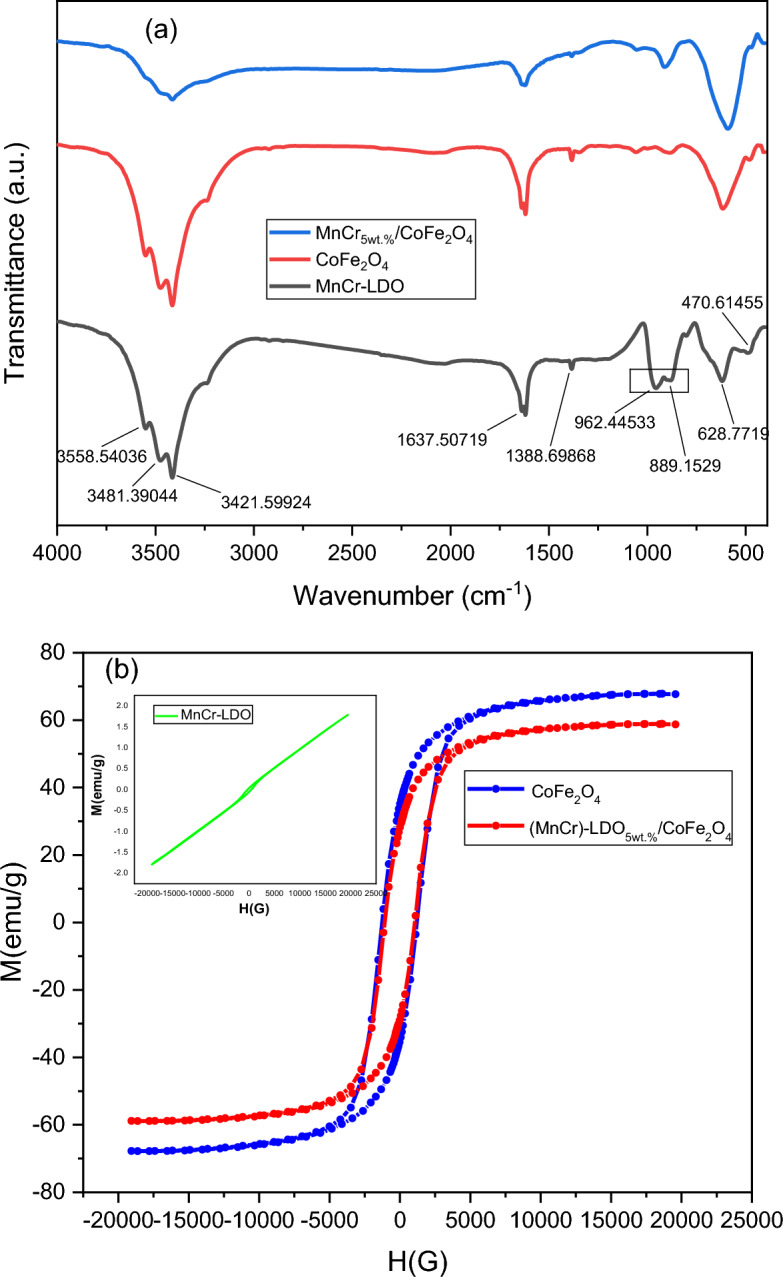
MnCr-LDO, CoFe_2_O_4_, and (MnCr)-LDO_5wt.%_/CoFe_2_O_4_ (**a**) FTIR spectra, and (**b**) VSM curves, inset shows VSM for MnCr-LDO.

Figure 6a shows the FTIR spectra of MnCr-LDO, CoFe_2_O_4_, and (MnCr)-LDO_5wt.%_/CoFe_2_O_4_ samples. The composite has an essentially similar FTIR spectrum to that of MnCr-LDO and CoFe_2_O_4_. A broad band appeared in the wavenumber range of 3558–3421 cm^−1^. This broad band was ascribed to OH− groups^38^.The intensity of this band was reduced for the composite sample, as shown in Fig. 5a. Peaks at 1637 cm^−1^ were attributed to H_2_O molecule vibrations. This peak decreased for the composite sample. This reduction is mainly connected with the heat treatment performed on the composite sample which can result in water evaporation. The peak at 1388 cm^−1^ showed the vibration mode of CO_3_^2−^^39^. The lower band frequencies between 1000 and 400 cm^−1^ correspond to the lattice vibration of metal–oxygen bonding to the layers of the LDO and CoFe_2_O_4_^38^. The vibration modes of the carbonate anions were also illustrated at 962 cm^−1^ peak^38^. The presence of such vibrations reveals the intercalation of carbonate ions into the interlayer space of MnCr-LDO. Figure 6b shows the VSM curves for the prepared samples. It is clearly seen, from the inset of Fig. 6b, that MnCr-LDO has dominated paramagnetic behavior coupled with weak ferromagnetic behavior that has been seen from the tiny hysteresis loop appeared in the low field region. On the other hand, the strong ferromagnetic nature of the pure CoFe_2_O_4_ and the (MnCr)-LDO_5wt.%_/CoFe_2_O_4_ samples were confirmed by the large hysteresis loops which are recorded as fingerprint for hard ferrites materials.

### XPS analysis

**Figure 7:**
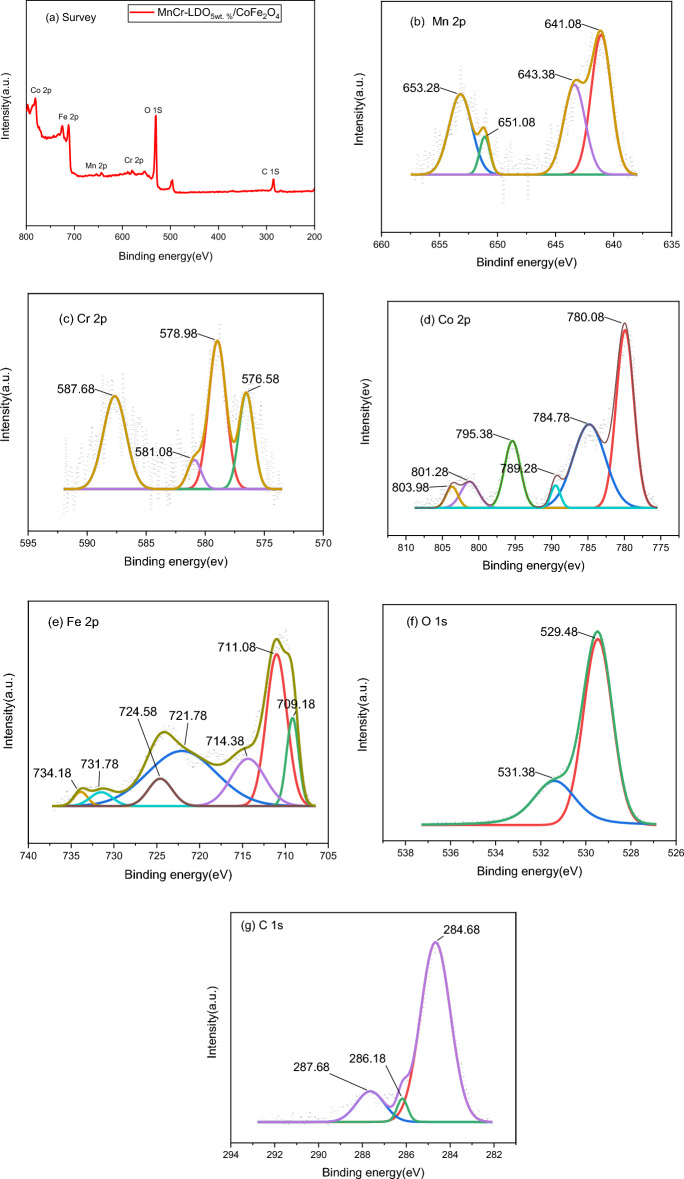
XPS spectrum for (MnCr)-LDO_5wt.%_/CoFe_2_O_4_ nanocomposite (**a**) Survey data (**b**) Mn 2p (**c**) Cr 2p (**d**) Co 2p (**e**) Fe 2p (f) O 1s and (g) C 1s.

X-ray photoelectron spectroscopy (XPS) studies were carried out to examine the surface chemical composition and oxidation state of the produced (MnCr)-LDO_5wt.%_/CoFe_2_O_4_ nanocomposite. The wide range survey XPS spectrum (Fig. 7a) confirmed the elemental existence of Mn, Cr, Co, Fe, O and C in the prepared (MnCr)-LDO_5wt.%_/CoFe_2_O_4_ nanocomposite. Two spin–orbit doublets were used to match the Mn 2p core-level spectra (Fig. 7b). The Mn^2+^ 2p_3/2_ and Mn^2+^ 2p_1/2_ peaks are situated at 641.08 eV and 651.08 eV, respectively, with their satellites at 643.38 eV and 653.28 eV^23,24^. Figure 7c shows the Cr 2p spectrum. The Cr^3+^ 2p_1/2_ and Cr^3+^ 2p_3/2_ have two wide peaks at 587.68 eV and 576 eV, respectively^3^. The binding energies of Cr 2p at 578.98 eV and 576.58 eV can be assigned to the Cr–OH and Cr–O, respectively^25^. The XPS spectrum of Co 2p (Fig. 7d) depicted two main peaks at ∼ 795.38 and ∼ 780.08 eV, which correspond to Co^2+^ 2p_1/2_and Co^2+^ 2p_3/2_, respectively^30^. Co 2p satellites appeared at 784.78, 789.28, 801.28 and 803.98 eV. Moreover, the spectrum of Fe^3+^ 2p (Fig. 7e) exhibits strong peaks located at around 724.58 and 7011.08 eV, which are assigned to Fe^3+^ 2p_1/2_ and Fe^3+^ 2p_3/2_, respectively, indicating a 3+ valency of Fe ions [31–34]. In the O 1s spectrum (Fig. 7f), the binding energy peaks are located at 529.48 and 531.38, corresponding to the characteristic bands of oxygen in the M–O lattice, surface hydroxyl group and bound water, respectively^25^. The C 1s spectrum (Fig. 7g) can be deconvoluted into three main peaks with the highest binding energy at 287.68 eV corresponding to O–C=O in CO_3_^2−^. This further confirms the existence of CO_3_^2−^ in the interlayer of MnCr-LDO. As a result, XPS investigations clearly supported the production of the (MnCr)-LDO_5wt.%_/CoFe_2_O_4_ nanocomposite.

### Adsorption analysis

#### Effect of contact time

**Figure 8:**
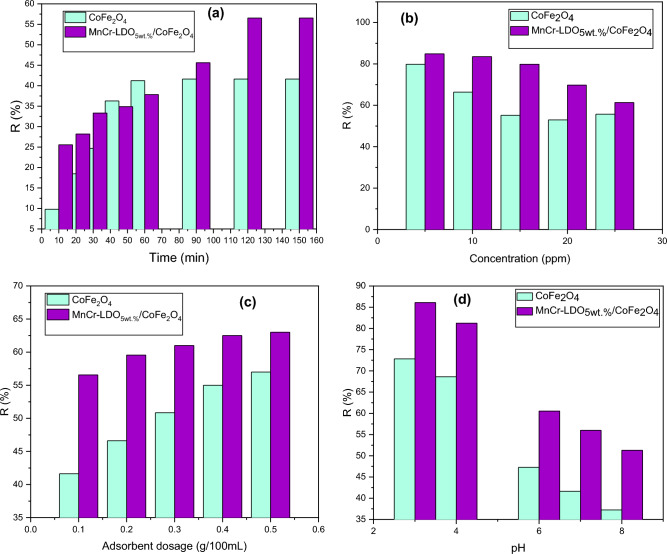
MO removal percentage R (%) by CoFe_2_O_4_ and (MnCr)-LDO_5wt.%/_CoFe_2_O_4_ nano-adsorbents versus (**a**) contact time (**b**) dye concentration, (**c**) adsorbent dosage and (**d**) pH.

One of the essential factors in adsorption processes is contact time, and it is crucial to build procedures for industrial applications that are both efficient and affordable. In order to better understand this, the impacts of contact time on the adsorption of MO by CoFe_2_O_4_ and (MnCr)-LDO_5wt.%_/CoFe_2_O_4_ adsorbents were investigated. Figure 8a shows MO removal percentage at different contact times (10–150 min) for 25 ppm dye concentration. As a primary impression, (MnCr)-LDO_5wt.%_/CoFe_2_O_4_ nanoadsorbent recorded higher MO removal percentages than pure CoFe_2_O_4_ for most applied contact times. This result is mainly due to the presence of MnCr-LDO inside the matrix of CoFe_2_O_4_ which offered more active vacant sites for the adsorption process owing to its layered structure. Also, it can be seen, from Fig. 8a, that the adsorption was particularly quick at first due to the nanoadsorbents' high number of unoccupied active sites. After a while, adsorption gradually increased due to increasing saturation of the active sites, until it reached a flat plateau, i.e. equilibrium region^40^. For CoFe_2_O_4_, increasing contact time increased MO dye adsorption until it remained constant after 60 min with a maximum percentage of dye removal of 41.61%. On the other hand, increasing contact time using (MnCr)-LDO_5wt.%_/CoFe_2_O_4_, increased MO dye adsorption to reach 56.64%, after that it remained constant at contact time ˃ 120 min, which is higher than that of CoFe_2_O_4_.

#### Effect of initial dye concentration

To overcome the mass transfer resistance of metal ions between the aqueous and solid phases, the initial concentration of MO dye is crucial. Both CoFe_2_O_4_ and (MnCr)-LDO_5wt.%_/CoFe_2_O_4_ were used to study the sorption of MO dye at concentrations ranging from 5 to 25 ppm. Figure 8b indicates clearly that the percentage of removal for both nano-sorbents decreases with increasing concentration. When the concentration of MO increased, the efficiency with which they could be removed from the sport's surface diminished. More binding sites are accessible when the concentration of MO is low to begin with. The number of ions vying for biomass binding sites, however, increased as concentration rose^42^.

#### Effect of adsorbent dosage

One of the most critical parameters for rapid and efficient dye removal is the size and amount of adsorbent, which must be optimized. The adsorbent dosage is an important parameter in adsorption studies because it determines the capacity of the adsorbent for a given initial concentration of dye solution. The effect of the adsorbent dosage was studied by varying the sorbent amounts from 0.1 to 0.5 g/100 mL. This effect was studied at an initial concentration of 25 ppm of MO dye. It may be observed from Fig. 8c that the removal percentage of MO increased rapidly with the increase in adsorbent dosage until 0.5 g/100 mL for both nano sorbents. This can be attributed to increase adsorbent surface area and the availability of more adsorption sites with increasing adsorbent dosage. For the same amount of nanosorbent (0.5 g/100 mL), the percentage of dye removal using CoFe_2_O_4_ was 55%, which is lower than that obtained using (MnCr)-LDO_5wt.%_/CoFe_2_O_4_ which is 65%.

#### Effect of pH

Solution pH plays an important role in dye adsorption. Figure 8d displays the effect of solution pH on MO dye adsorption. 25 ppm of MO solutions were agitated with 0.1 g/100 mL of both CoFe_2_O_4_ and (MnCr)-LDO_5wt.%_/CoFe_2_O_4_ at different pH values (3–8) at 25 °C. A decrease in the removal percentage of MO dye was found with increasing pH for both nanosorbents. Moreover, the increase in the dye removal percentage is higher for (MnCr)-LDO_5wt.%_/CoFe_2_O_4_ (86.1%) than that for CoFe_2_O_4_ (72.8%) at pH = 3. Due to the electrostatic attraction that occurs when the pH is 3, positively charged surface sites on the adsorbent favor the adsorption of dye anions. As a result, the adsorption capacity in an acidic medium is high for MO due to excess positive sites arises from acid activation. The adsorption capacity decreases when pH ˃ 3 as a result of OH groups and MO anionic ions adsorption competition on the active sites^24^. Consequently, CoFe_2_O_4_ and (MnCr)-LDO_5wt.%_/CoFe_2_O_4_ capacities for MO removal were optimized by many factors, i.e., acidic media (pH = 3) showed good results but increasing MO dye initial concentration decreased the removal % of the dye for both adsorbents.

#### Adsorption isotherms

The adsorption isotherm is a correlation between the quantity of adsorbate (MO dye) taken from the liquid phase per unit mass of adsorbent at a constant temperature and pH. The development of an adsorption system necessitates the use of adsorption isotherms. For accurate prediction of adsorption parameters and quantitative comparison of adsorption behavior across various adsorbent systems, a thorough mathematical description of equilibrium adsorption capacity is crucial. Parameters from equilibrium isotherms may shed light on the adsorbent's surface characteristics, affinity, and sorption process. The optimal conditions for developing adsorption systems depend on identifying the best correlation coefficients (R^2^) of equilibrium curves. The equilibrium data were examined using three isotherms termed; Langmuir, Freundlich, and Temkin. These isotherm models were represented according to the following relations^41,42^:

8 $$Langmuir\; isotherm: \frac{1}{{q_{e} }} = \frac{1}{{q_{m} }} + \frac{1}{{K_{L} q_{m} }}\frac{1}{{C_{e} }},$$

9 $$Freundlich\; isotherm: \ln q_{e} = \ln K_{F} + \frac{1}{{n_{F} }} \ln C_{e} ,$$

10 $$Temkin\; isotherm: q_{e} = q_{m} \ln k_{T} + q_{m} \ln C_{e} ,$$

where q_m_ represents the maximum amount of adsorbed dye, q_e_ and C_e_ defined as previously mentioned in Table 1, K_L_ represents the Langmuir constant, K_F_ and n_F_ represent the Freundlich constants, and k_T_ is the Temkin constant.

**Figure 9:**
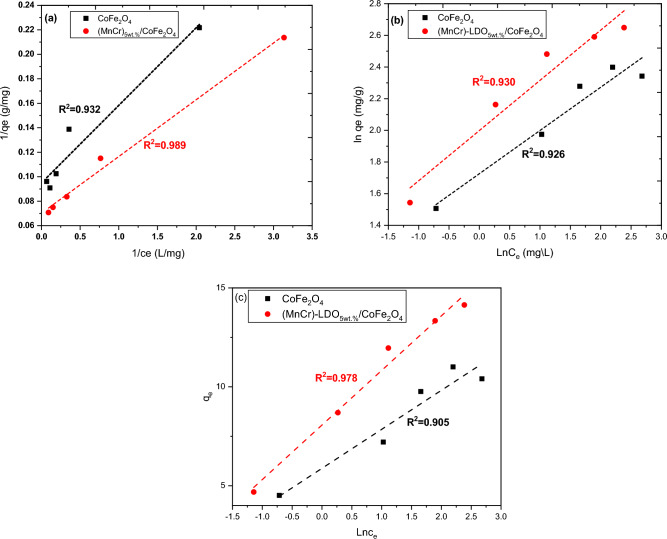
CoFe_2_O_4_ and (MnCr)-LDO_5wt.%_/CoFe_2_O_4_ (**a**) Langmuir model (**b**) Freundlich model and (**c**) Temkin model.

The Langmuir model states that monolayer adsorption occurs on an adsorbent with a surface that is structurally homogeneous. The binding sites in this instance have a similar propensity for adsorption^43^. Linear plots of $\frac{1}{{C}_{e}}$ versus $\frac{1}{{q}_{e}}$ are shown in Fig. 9a for CoFe_2_O_4_ and (MnCr)-LDO_5wt.%_/CoFe_2_O_4_ nanoadsorbents. The lines were fitted according to Langmuir model, Eq. (8), and the fitting parameters (q_m_ and K_L_) and correlation coefficients (R^2^) were easily determined and listed in Table 3 for both nanoadsorbents. As seen from Table 3, the correlation coefficient (R^2^) in case of (MnCr)-LDO_5wt.%_/CoFe_2_O_4_ is higher than that of CoFe_2_O_4_. On the other hand, the maximum monolayer adsorption capacity for dye removal was q_m_ = 14.258 mg/g and Langmuir constant was K_L_ = 1.512 for (MnCr)-LDO_5wt.%_/CoFe_2_O_4_ and q_m_ = 10.537 mg/g and K_L_ = 1.497 for CoFe_2_O_4_. The essential properties of the Langmuir isotherm may be described by the dimensionless constant R_L_, also known as the equilibrium parameter. It may be computed using the equation below^44^:

11 $$R_{L} = \frac{1}{{1 + K_{L} C_{o} }}$$

**Table 3:** Isotherm constant parameters and correlation coefficients calculated for adsorption of MO onto CoFe_2_O_4_ and (MnCr)-LDO_5wt.%_/CoFe_2_O_4_ adsorbents.

Isotherms	R^2^		Fitting parameters	
	CoFe_2_O_4_	(MnCr)-LDO_5wt.%_/CoFe_2_O_4_	CoFe_2_O_4_	(MnCr)-LDO_5wt.%_/CoFe_2_O_4_
Langmuir	0.932	0.989	$q_{m}$ = 105.37 mg/g K_L_ = 1.497 R_L_ = 0.026	$q_{m}$ = 142.58 mg/g K_L_ = 1.512 R_L_ = 0.025
Freundlich	0.926	0.930	K_F_ = 5.619 n_f_ = 3.663	K_F_ = 7.380 n_f_ = 3.154
Temkin	0.905	0.978	q_m_ = 1.971 K_T_ = 19.708	q_m_ = 2.758 K_T_ = 18.625

By substituting for K_L_ and the initial dye concentration C_o_, the equilibrium parameters R_L_ were estimated and were listed in Table 3 for both nanoadsorbents. As a result, the nature of the adsorption process may be characterized by the value of R_L_ as being un-favorable for R_L_ greater than unity, linear for R_L_ equals unity, favorable for R_L_ greater than zero and less than unity, or irreversible for R_L_ equals zero^44^ Table 3 indicated that both adsorbents have R_L_ values between 0 and 1, suggesting that MO dye is adsorbed to them favorably.

The empirical Freundlich model posits that the more strongly bound sites are used up first and that the binding strength diminishes with the increasing degree of site occupancy; this is done on the basis of the multilayer development of adsorbate on the heterogeneous solid surface of the adsorbent^45^. The plots of lnq_e_ aganist lnC_e_ for the adsorption of MO onto CoFe_2_O_4_ and (MnCr)-LDO_5wt.%_/CoFe_2_O_4_ are shown in Fig. 9b. The intercept and slope of the linear plots reflect the extent of adsorption and the degree of nonlinearity between the dye solution concentration and the adsorption, respectively. The Freundlich parameters (${n}_{F},$ K_F_, and R^2^) are listed in Table 3. The value of n_F_ varies with the heterogeneity of the adsorbent, and for a favorable adsorption process, the value of n_F_ should be less than 10 and higher than unity. As can be seen from Table 3 that the value of n_F_ and K_F_ obtained was 3.663 and 5.619 for CoFe_2_O_4_, and 3.154 and 7.380 for (MnCr)-LDO_5wt.%_/CoFe_2_O_4_ indicating a favorable adsorption process. The less than unity values of $\frac{1}{{n}_{F}}$ referred to satisfactory adsorption process. The values of correlation coefficients (R^2^) are 0.926 and 0.930 for CoFe_2_O_4_ and (MnCr)-LDO_5wt.%_/CoFe_2_O_4_, respectively. These R^2^ values showed that Freundlich isotherm was not proper to designate the data of the experiment.

**Figure 10:**
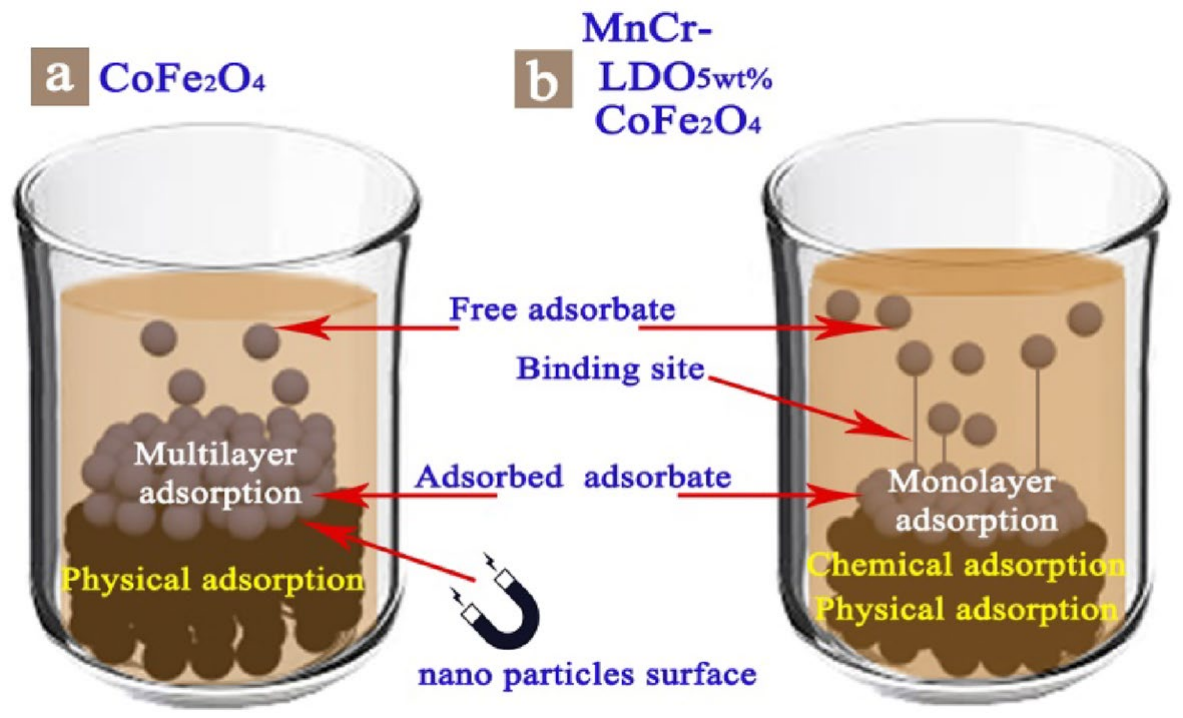
Schematic representation for MO adsorption by (**a**) CoFe_2_O_4_ and (**b**) (MnCr)-LDO_5wt.%_/CoFe_2_O_4_.

Despite the Langmuir and Freundlich isotherms, which assume that sorption free energy is independent of surface coverage, the Temkin isotherm model takes into consideration interactions between adsorbents and metal ions to be adsorbed^46–48^. Figure 9c shows the plot of q_e_ versus ln C_e_ for CoFe_2_O_4_ and (MnCr)-LDO_5wt.%_/CoFe_2_O_4_ nanoadsorbents. The data were linearly fitted according to Temkin model (Eq. 10). Temkin parameters (b_T_ and K_T_) were determined from the slope and intercept of the plots and their values together with R^2^ were listed in Table 3. The applicability of the isotherm equation to describe the adsorption process was judged by the correlation coefficients, R^2^ values. The adsorption isotherm models fitted the data in the order of:

Langmuir ~ Freundlich > Temkin in case of CoFe_2_O_4_ suggested the homogenous distribution of active sites beside increasing the probability of formation multilayers of MO dye on to the magnetic CoFe_2_O_4_ nanoadsorbent so that the first charged adsorbent layer hinder from multiple layer adsorption, the whole process is schematically shown in Fig. 10a. The value of R^2^ is low (0.905) in the case of CoFe_2_O_4_, indicating poor agreement and efficiency of the Temkin isotherm in interpreting the experimental data. For (MnCr)-LDO_5wt.%_/CoFe_2_O_4_ the order is Langmuir > Temkin > Freundlich. The above order revealed that the equilibrium data are better fitted by the three-parameter models in case of (MnCr)-LDO_5wt.%_/CoFe_2_O_4_ but for CoFe_2_O_4_ two-parameter models were better fitted. In case of (MnCr)-LDO_5wt.%_/CoFe_2_O_4_ the Langmuir equation represents the adsorption process very well (R^2^ > 0.98) addition to Temkin (R^2^ > 0.97). The fact that the Langmuir isotherm fits the experimental data very well may be due to homogenous distribution of active sites and the formation of monolayer of MO dye over the magnetic (MnCr)-LDO_5wt.%_/CoFe_2_O_4_nanoadsorbent, as shown in Fig. 10b. Table 4 displays the maximum adsorption capacities for adsorbents reported in other literatures.

**Table 4:** comparison of maximum MO adsorption capacity with other adsorbents.

Adsorbent	Optimum pH	q_m_ (mg/g)	References
MgNiAl-LDO	8.0	375.4	^49^
ZnAl-LDO	6.0	181.9	^50^
Activated carbon/NiFe2O4	3.0	182.8	^51^
ZnO-MgFe_2_O_4_/ZnAl-LDH	6.0	101	^52^
ZnO-MgFe_2_O_4_/MgAl-LDH	6.0	99.0	^52^
Co/MWCNTs	2.0	170.0	^53^
Graphene oxide	3.0	16.83	^54^
Chitosan/Al_2_O_3_/magnetite	4.5	446.2	^55^
CoFe_2_O_4_	3.0	105.3	Current study
(MnCr)-LDO_5wt.%_/CoFe_2_O_4_	3.0	142.5	Current study

#### Adsorption kinetics

Adsorption kinetics research is useful in estimating adsorption rates and mechanisms^56^. The kinetics of MO adsorption onto the synthesized nanoparticles was examined using four kinetic models: pseudo-first order, pseudo-second order, Elovich, and intra-particle diffusion.

The pseudo-first-order model indicates that physical adsorption is the dominant process. The linearized pseudo-first-order equation is given by the following relation^57^:

12 $$\log \left( {q_{e} - q_{t} } \right) = \log q_{e} - k_{1} t,$$

where q_e_ and q_t_ are previously defined in Table 1, while k_1_ (min^−1^) is the rate constant.

**Figure 11:**
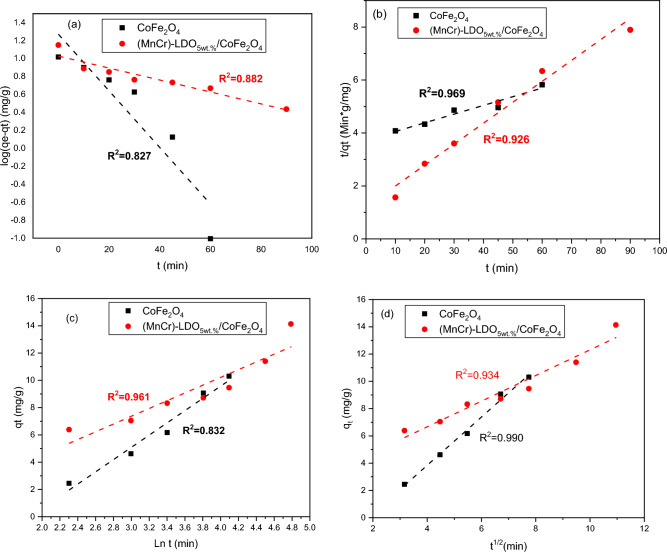
CoFe_2_O_4_ and (MnCr)-LDO_5wt.%_/CoFe_2_O_4_ (**a**) pseudo-first order model (**b**) pseudo-second order model, (**c**) Elovich model, and (**d**) intra-particle diffusion model.

Figure 11a depicts the experimental adsorption data obtained for different time interval at initial concentration of 25 ppm MO dye solution fitted to pseudo-first order kinetic model. The model parameters (k_1_ and q_e_) were determined from the slope and the intercept and were listed in Table 5. The low value of correlation coefficients for CoFe_2_O_4_ and (MnCr)-LDO_5wt.%_/CoFe_2_O_4_ adsorbents and the non-reasonable difference between the experimental and calculated adsorption capacity (q_e_) of first-order kinetic showed that this model fail to interpret the experimental data.

**Table 5:** Kinetic parameters for the adsorption of MO onto CoFe_2_O_4_ and (MnCr)-LDO_5wt.%_/CoFe_2_O_4_ adsorbents.

Isotherms	R^2^		Parameters	
	CoFe_2_O_4_	(MnCr)-LDO_5wt.%_/CoFe_2_O_4_	CoFe_2_O_4_	(MnCr)-LDO_5wt.%_/CoFe_2_O_4_
Pseudo-first order	0.827	0.882	$k_{1}$ = 0.0726 min^−1^	$k_{1}$ = 0.015 min^−1^
Pseudo-second order	0.926	0.969	k_2_ = 0.0003 (g/mg.min)	k_2_ = 0.005 (g/mg.min)
Elovich	0.961	0.832	α = 0.694 (mg/g.min) β = 0.223 (g/mg)	α = 1.885 (mg/g.min) β = 0.351 (g/mg)
Intraparticle diffusion	0.990	0.934	k_int_ = 1.771 C = − 3.238	k_int_ = 0.941 C = 2.904

According to the pseudo-second order model, chemisorption is the main phenomenon. Electron sharing or electron transfer between adsorbent and adsorbate occurs in this process^56^. The following equation gives the mathematical expression for the model as follows^58^

13 $$\frac{t}{{q_{t} }} = \frac{1}{{k_{2} q_{e}^{2} }} + \frac{t}{{q_{e} }}$$

where k_2_ is the rate constant of the pseudo-second-order kinetic equation and q_t_, q_e_, and t are the same as defined in Table 1. Linear graphs are obtained from the plot of $\frac{t}{{q}_{t}}$ against t as shown in Fig. 11b. The fitting parameters (q_e_ and k_2_) are calculated from the slope and the intercept. Adsorption experiments for CoFe_2_O_4_ and (MnCr)-LDO_5wt.%_/CoFe_2_O_4_ were found to be consistent with a pseudo-second order kinetic model, with rate constants of 3 × 10^−4^ (g/mg.min) and 50 × 10^−4^ (g/mg.min), respectively, as shown by the experimental results in Table 5.

The Elovich model may be used to characterize second order kinetics and is typically applicable for systems with a heterogeneous adsorbing surface^58^. The Elovich model equation does not suggest a specific mechanism for the adsorption process^59^. The following equation gives the model's linear form as follows^60^:

14 $$q_{t} = \frac{1}{\beta }\ln \left( {\alpha \beta } \right) + \frac{1}{\beta }{\text{ln}}\left( {\text{t}} \right),$$

where α (mg/g. min) is the initial adsorption rate, while β (g/mg) represents the amount of surface coverage and is the activation energy of chemisorption. These coefficients were obtained from the plot of q_t_ versus ln t, as shown in Fig. 11c, and were tabulated in Table 5. The activation energy for chemisorption (g/mg) and the amount of surface covering β are responsible for the initial sorption rate α (mg/g.min). Adsorption of MO by CoFe_2_O_4_ follows the Elovich model, as shown by high R^2^ value (0.961). On the other hand it is not suitable for describing experimental data for (MnCr)-LDO_5wt.%_/CoFe_2_O_4_ adsorbent (R^2^ = 0.832).

The intra-particle diffusion model is commonly used for porous materials, and the physical adsorption phenomenon involves the diffusion of adsorbate into pores of varying sizes. Diffusion via intra-particle diffusion is the rate-limiting step in the transfer of adsorbate molecules/ions from the bulk solution to the adsorbent solid surface. The following equation represents the diffusion through the intraparticle diffusion model^60^:

15 $$q_{t} = k_{int} t^{{{\raise0.7ex\hbox{$1$} \!\mathord{\left/ {\vphantom {1 2}}\right.\kern-0pt} \!\lower0.7ex\hbox{$2$}}}} + C,$$

where k_int_ represents the intra-particle diffusion rate constant and C represents the intercept constant.

A plot of q_t_ versus t^1/2^ is shown for both nanoadsorbents in Fig. 11d. The experimental data were fitted according to intra-particle diffusion model. The slope, which is equivalent to q_t_, intercept, that gives C, and R^2^ values were tabulated in Table 5 for CoFe_2_O_4_, and (MnCr)-LDO_5wt.%_/CoFe_2_O_4_ nanoadsorbents. The results indicated that MO adsorption on CoFe_2_O_4_ obeyed the intra-particle diffusion model with high R^2^ values of 0.990. On the other hand, this model is not suitable in case of (MnCr)-LDO_5wt.%_/CoFe_2_O_4_ nanoadsorbent as it showed low R^2^ value of 0.934. These data imply that the physical adsorption process dominates MO removal via CoFe_2_O_4_ nanodsorbent, whereas both physical and chemical adsorption processes influence MO removal via (MnCr)-LDO_5wt.%_/CoFe_2_O_4_ nanodsorbent, as schematically illustrated in Fig. 10b.

#### Reusability analysis

**Figure 12:**
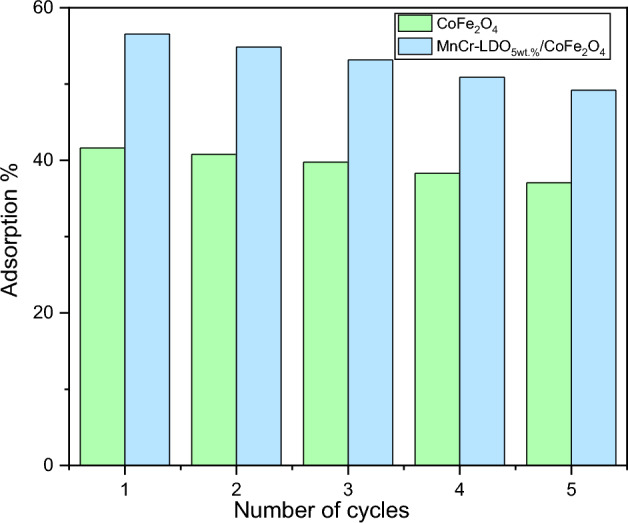
CoFe_2_O_4_ and (MnCr)-LDO_5wt.%_/CoFe_2_O_4_ recycling process.

A reuse study of CoFe_2_O_4_ and (MnCr)-LDO_5wt.%_/CoFe_2_O_4_ nanoadsorbents for MO adsorption was carried out in order to verify the possibility of regeneration and reuse of the adsorbent material, an important factor for the replicability and economy of the process. Thedesorption of methyl orange was investigated, and the results are shown in Fig. 12. The results presented in Fig. 12 demonstrated that CoFe_2_O_4_ and (MnCr-LDO)_5wt.%_/CoFe_2_O_4_ showed good adsorptive capacity even after five adsorption–desorption cycles. In all cycles, an external magnetic field was used to recover the adsorbent, thus reducing the abrupt mass losses that occur in other recovery processes of the adsorbent material.

#### Photocatalytic study

**Figure 13:**
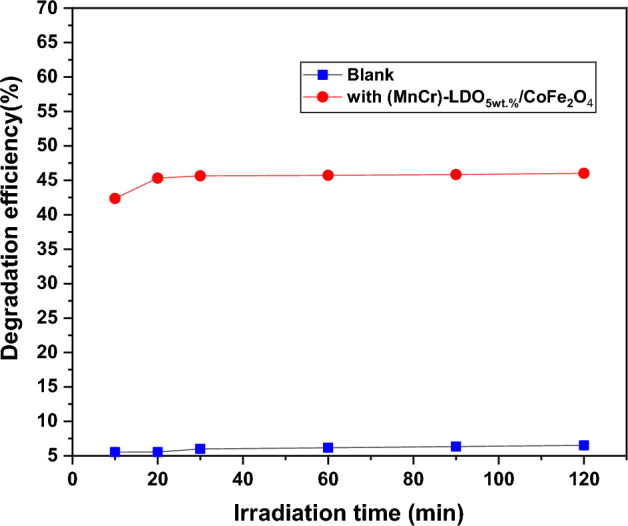
Photo degradation efficiency with irradiation time for blank and (MnCr)-LDO_5wt.%_/CoFe_2_O_4_ nanocomposite.

The degradation of MO under UV light was used to gauge the composite catalyst's photocatalytic activity. The removal efficiencies for blank and (MnCr)-LDO_5wt.%_/CoFe_2_O_4_ nanocomposite catalyst are shown in Fig. 13 as a function of irradiation time. About 46% of MO was decomposed on (MnCr)-LDO_5wt.%_/CoFe_2_O_4_ within 120 min, demonstrating the predicted performance of the composite in photocatalysis as compared to the poor degradation of MO in the blank test (6.5%). The following two aspects were probably responsible for the improvement of photocatalysis: First, photocatalytic processes depend greatly on adsorption capacity. Second, (MnCr)-LDO_5wt.%_/CoFe_2_O_4_ exhibits a unique linked nanostructure, i.e. the presence of MnCr-LDO affects the photocatalytic activity owing to the development of mixed metal oxide and their composites^20,23^. Additionally, any transferred charge carriers have a greater chance of becoming accessible to either oxidants or reductants in the solution^21,22^.

## Conclusions

In summary, a novel nanocomposite termed (MnCr)-LDO_5wt.%_/CoFe_2_O_4_ was successfully prepared via the co-precipitation route. Its removal efficiency for methyl orange dye was examined and compared to that of pure CoFe_2_O_4_. Several parameters have been examined to show their effect on the MO adsorption process. These parameters were the contact time, the dye concentration, the nanoadsorbent dosage, and pH. The findings showed that the maximum removal percentage was 86.1% for 25 ppm of MO by 0.1 g/100 mL of (MnCr)-LDO_5wt.%_/CoFe_2_O_4_ at pH = 3. Investigations of isotherms and kinetics were conducted under batch conditions. The Langmuir isotherm matched the experimental data for both nanoadsorbents, quite well due to the homogeneous distribution of active sites. Adsorption kinetics data were found to be compatible with intra-particle diffusion and pseudo-second order models for CoFe_2_O_4_ and (MnCr)_5wt.%_/CoFe_2_O_4_, respectively. These findings implied that MO removal through CoFe_2_O_4_ nanodsorbent is dominated by the physical adsorption process, while both physical and chemical adsorption processes were influencing MO removal by (MnCr)-LDO_5wt.%_/CoFe_2_O_4_ nanodsorbent. Furthermore, the magnetic characteristics of CoFe_2_O_4_ and (MnCr)-LDO_5wt.%_/CoFe_2_O_4_ aided in the reuse process by removing the requirement for time-consuming and expensive effective methods. A total of five adsorption–desorption cycles were performed to determine the prepared adsorbents’ recyclable nature.

## Data Availability

The data that support the findings of this study are available from the corresponding author upon reasonable request.
